# BCG+MMC trial: adding mitomycin C to BCG as adjuvant intravesical therapy for high-risk, non-muscle-invasive bladder cancer: a randomised phase III trial (ANZUP 1301)

**DOI:** 10.1186/s12885-015-1431-6

**Published:** 2015-05-27

**Authors:** Dickon Hayne, Martin Stockler, Steve P. McCombie, Venu Chalasani, Anne Long, Andrew Martin, Shomik Sengupta, Ian D. Davis

**Affiliations:** 1 Australian and New Zealand Urogenital and Prostate Cancer Trials Group (ANZUP), Camperdown, NSW 1450 Australia; 2 School of Surgery, University of Western Australia, Crawley, WA 6009 Australia; 3 Fiona Stanley Hospital Urology (proudly supported by WAURO), Murdoch, WA 6150 Australia; 4 NHMRC Clinical Trials Centre, University of Sydney, Sydney, NSW 2050 Australia; 5 Department of Urology, Hornsby Ku-Ring-Gai Hospital, Sydney, NSW 2077 Australia; 6 Department of Urology, Austin Health, Melbourne, VIC 3084 Australia; 7 Austin Department of Surgery, University of Melbourne, Melbourne, VIC 3010 Australia; 8 Ludwig Institute for Cancer Research, Austin Hospital, Melbourne, VIC 3084 Australia; 9 Monash University Eastern Health Clinical School, Box Hill, Melbourne, VIC 3128 Australia

**Keywords:** Bladder cancer, Transitional cell carcinoma, Intravesical, BCG, Mitomycin

## Abstract

**Background:**

Despite adequate trans-urethral resection of the bladder tumour (TURBT), non-muscle-invasive bladder cancer (NMIBC) is associated with high rates of recurrence and progression. Instillation of Bacillus Calmette-Guérin (BCG) into the urinary bladder after TURBT (adjuvant intravesical administration) reduces the risk of both recurrence and progression, and this is therefore the standard of care for high-risk tumours. However, over 30 % of people still recur or progress despite optimal delivery of BCG. Our meta-analysis suggests that outcomes might be improved further by using an adjuvant intravesical regimen that includes both mitomycin and BCG. These promising findings require corroboration in a definitive, large scale, randomised phase III trial using standard techniques for intravesical administration.

**Methods and design:**

The BCG + MMC trial (ANZUP 1301) is an open-label, randomised, stratified, two-arm multi-centre phase III trial comparing the efficacy and safety of standard intravesical therapy (BCG alone) against experimental intravesical therapy (BCG and mitomycin) in the treatment of adults with resected, high-risk NMIBC. Participants in the control group receive standard treatment with induction (weekly BCG for six weeks) followed by maintenance (four-weekly BCG for ten months). Participants in the experimental group receive induction (BCG weeks 1, 2, 4, 5, 7, and 8; mitomycin weeks 3, 6, and 9) followed by four-weekly maintenance (mitomycin weeks 13, 17, 25, 29, 37, and 41; BCG weeks 21, 33, and 45). The trial aims to include 500 participants who will be centrally randomised to one of the two treatment groups in a 1:1 ratio stratified by T-stage, presence of CIS, and study site. The primary endpoint is disease-free survival; secondary endpoints are disease activity, time to recurrence, time to progression, safety, health-related quality of life, overall survival, feasibility, and resource use.

**Trial registration:**

This trial is registered with the Australian New Zealand Clinical Trials Registry (ACTRN12613000513718).

## Background

### Bladder cancer

Bladder cancer is a major global cause of suffering and mortality. Worldwide, it is among the top 10 cancers in terms of incidence and mortality with an estimated 380,000 cases and 150,000 deaths in 2008 [1]. Contrary to trends for most other cancers, five-year survival from bladder cancer has reduced internationally over the last 20 to 30 years [2, 3].

Bladder cancers are classified according to their grade (high or low) and according to their depth of penetration through the bladder wall [4]. Muscle-invasive bladder cancer includes all tumours that invade into detrusor muscle or beyond (T2-4). Non-muscle-invasive bladder cancer (NMIBC) includes carcinoma-in-situ (CIS), papillary tumours (Ta), and tumours that invade into lamina propria but not detrusor muscle (T1). Whilst NMIBC is often treated successfully with transurethral resection of the bladder tumour (TURBT), it can be associated with a high risk of recurrence and progression (increase in grade or stage). These risks can be estimated based on the presence, or absence, of certain prognostic indicators [5]. In people with high-risk NMIBC (high-grade Ta, or any grade T1), the five-year risk of recurrence ranges from 46-78 % and the five-year risk of progression ranges from 6-45 % depending on further risk stratification [5]. Disease progression includes the development of muscle-invasive disease, which is associated with a poorer prognosis [6] and usually warrants radical treatment with cystectomy (removal of the urinary bladder) or irradiation. Various intravesical therapies (instillation of agents into the urinary bladder) have been used in the treatment of NMIBC in an attempt to reduce the risk of recurrence and progression.

### Intravesical Bacillus Calmette-Guérin (BCG)

Intravesical BCG is thought to work by stimulating an antigen-mediated immune response against bladder tumour cells. Meta-analyses have demonstrated that intravesical BCG after TURBT (adjuvant BCG) significantly reduces both the risk of recurrence (odds ratio 0.3, 95 % CI 0.21-0.43 [7]) and progression (odds ratio 0.73, 95 % CI 0.6-0.89 [8]) of NMIBC, as compared with TURBT alone. The reduced risk of progression is only evident if the adjuvant BCG treatment includes both an induction course (usually weekly for four-to-six weeks) and a subsequent maintenance course (less frequent instillations over the following months or years) [8]. In view of the significant toxicities that can occur with intravesical BCG, its use is currently not recommended for low-risk NMIBC [4, 9]. Despite optimal delivery of adjuvant BCG, long-term failure rates of 30-40 % are encountered [10].

### Intravesical chemotherapy

Instillation of chemotherapeutic drugs into the bladder after TURBT (adjuvant intravesical chemotherapy) is another strategy that is employed in the treatment of NMIBC, and several different agents have been shown to be effective. For example, adjuvant intravesical mitomycin including an initial and maintenance instillations has been shown to reduce five-year recurrence rates from 60 % to 37 % (p < 0.001) [11]. A meta-analysis of EORTC and MRC randomised clinical trials has demonstrated that adjuvant intravesical chemotherapy is effective in reducing recurrence (hazard ratio 0.8, 95 % CI 0.72-88), but not progression or overall survival [12].

### Combination intravesical treatment (BCG and chemotherapy)

Our recent meta-analysis of all published randomised trials examining the addition of intravesical chemotherapy to BCG suggests that it may well be beneficial in people with Ta or T1 disease, but not in those with CIS alone [13]. When the single trial that included participants with CIS alone was excluded, the addition of chemotherapy to BCG was found to reduce both recurrence (relative risk 0.75; 95 % CI 0.61 - 0.92) and progression (relative risk 0.45; 95 % CI 0.25-0.81), with no apparent increase in treatment toxicity [13]. However, the only individual randomised trial included in this meta-analysis that demonstrated significant improvements in recurrence and progression with the addition of chemotherapy to BCG was a randomised phase II trial reported by Di Stasi and colleagues [14].

The Di Stasi trial, including 212 participants with resected T1 tumours, followed for a median of 88 months, found that combined treatment with BCG and mitomycin improved recurrence rates (42 % vs. 58 %, p = 0.001), progression rates (9 % vs. 22 %, p = 0.045), disease-free survival (median of 69 vs. 21 months, p = 0.001), overall mortality (22 % vs. 32 %, p = 0.045), and disease-specific mortality (6 % vs. 16 %, p = 0.01) compared with BCG alone [14]. However, this trial did not include people with Ta disease, and mitomycin was administered using ‘electromotive delivery’ (an electric current passed through the catheter to plates on the skin of the lower abdominal wall) - a technique that has not been widely accepted or adopted. Therefore while these results are encouraging regarding the benefits of combined treatment with intravesical BCG and mitomycin in high-risk NMIBC, it remains unproven whether combination treatment is effective in all subgroups, or when treatment is delivered in a traditional way (without electromotive delivery).

### Pilot study

**Fig. 1:**
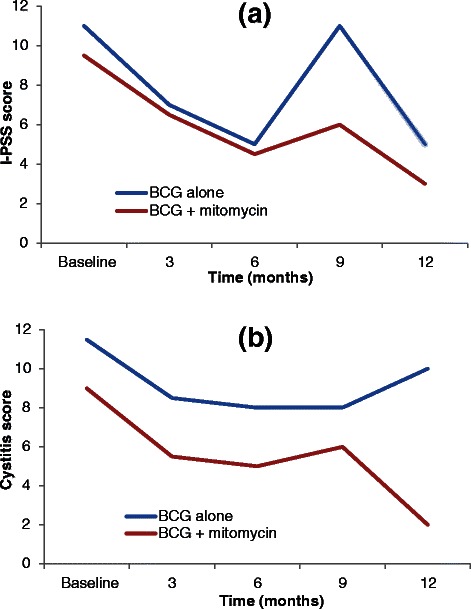
Pilot data on median (**a**) I-PSS and (**b**) cystitis scores

Our single-centre, non-randomised, pilot study has established the feasibility of adding mitomycin to BCG (without electromotive delivery) as adjuvant intravesical therapy for resected, high-risk NMIBC [15]. This pilot study involved 23 participants (11 BCG and mitomycin, 12 BCG alone) and assessed number of missed doses, treatment completion (number having received 75 % or more of planned doses), and participant-rated urinary symptoms. Participant-rated urinary symptoms were assessed using the I-PSS [16] and a cystitis score [17] at baseline, three months, six months, nine months, and one year. No induction treatment doses were missed for either group (0/99 BCG and mitomycin, 0/72 BCG alone) and 20 % or fewer maintenance doses were missed for both groups (8/99 BCG and mitomycin 24/120 BCG alone). Treatment completion exceeded 80 % for both groups (10/11 BCG and mitomycin, 10/12 BCG alone). Failure to complete treatment was due to one participant (BCG and mitomycin) progressing during maintenance treatment and requiring cystectomy, and two participants (both BCG alone) declining maintenance treatment. Of additional note, one participant (BCG and mitomycin) developed a reactive arthritis after induction and therefore completed scheduled mitomycin maintenance only. As can be seen in Fig. 1, combined treatment with BCG and mitomycin did not result in worse participant-rated urinary symptom scores at any interval during the first year, when compared to scores in those undergoing treatment with BCG alone.

## Methods and design

### Protocol overview

This is an open-label, randomised, stratified, two-arm multi-centre phase III trial comparing the efficacy and safety of standard intravesical therapy (BCG alone) against experimental intravesical therapy (BCG and mitomycin) in the treatment of adults with resected, high-risk NMIBC. Participants are randomised centrally to one of the two treatment groups in a 1:1 ratio stratified by T-stage, presence of CIS, and study site. It is an open-label trial because of differences in the appearance, scheduling, and handling of BCG and mitomycin. The trial is conducted by ANZUP in collaboration with the NHMRC Clinical Trials Centre, The University of Sydney. It is supported by funding from Cancer Australia, and is registered with the Australian New Zealand Clinical Trials Registry. Central ethical approval has been obtained from the Sydney Local Health District Ethics Review Committee (RPAH zone, HREC/13/RPAH/225). The study has also been approved by the Western Australian South Metropolitan Health Service Human Research Ethics Committee. Local ethical approval has been obtained for all participating centres, which currently includes the following Australian sites: Sydney Adventist Hospital, Northern Cancer Institute, Concord Repatriation General Hospital, Monash Medical Centre, Royal Melbourne Hospital, Austin Health, The Alfred Hospital, Footscray Hospital, Box Hill Hospital, Frankston Hospital, and Fiona Stanley Hospital.

### Inclusion criteria

1. Males or females with confirmed high-risk NMIBC (high-grade Ta, or any grade T1) on initial or re-resection histology (concurrent CIS is allowed)
2. Aged 18 years or older
3. TURBT performed within eight weeks prior to the date of randomisation with no remaining macroscopically visible disease post-resection
4. ECOG performance status of two or less [18]
5. Adequate bone marrow, renal, and liver function
6. Study treatment both planned and able to start within four weeks of randomisation
7. Has completed the health-related quality of life questionnaires or is unable to complete them because of literacy, insufficient English, or limited vision
8. Willing and able to comply with all study requirements, including treatment, timing and nature of all required assessments
9. Signed, written informed consent

### Exclusion criteria

1. Contraindications or hypersensitivity to investigational products, BCG and mitomycin
2. Prior treatment with any other intravesical agent including BCG or mitomycin (apart from single doses given post-TURBT)
3. Current or past transitional cell carcinoma of the upper urinary tract
4. Prior muscle-invasive transitional cell carcinoma of the bladder
5. Bladder dysfunction precluding intravesical therapy (e.g. severe urinary incontinence, bladder overactivity, or bladder spasticity)
6. Life expectancy less than three months
7. Congenital or acquired immune deficiencies, whether due to a concurrent disease, immunosuppressive therapy, or cancer therapy
8. Prior radiotherapy of the pelvis
9. Prior or current treatment with radiotherapy-response or biological-response modifiers
10. Clinical evidence of existing active tuberculosis
11. History of another malignancy within five years prior to registration (apart from non-melanomatous carcinoma of the skin)
12. Serious medical or psychiatric conditions that might limit the ability of the patient to comply with the protocol
13. Pregnancy, lactation, or inadequate contraception. Women must be post-menopausal, infertile, or use reliable means of contraception. Women of childbearing potential must have a negative pregnancy test done within seven days prior to registration. Men must have been surgically sterilised or use a (double if required) barrier method of contraception.

### Study treatments

**Fig. 2:**
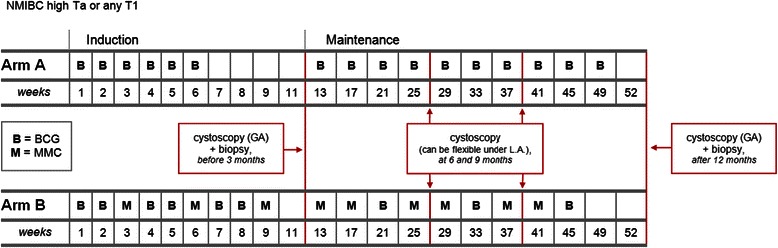
Summary of standard (Arm A) and experimental (Arm B) treatment regimens (displays treatment administration in terms of nominal weeks, and not calendar weeks, and thus accommodates treatment delays)

The two treatment regimens use the same (or equivalent) doses and schedule of BCG and mitomycin as the two arms in Di Stasi’s randomised trial, but without ‘electromotive delivery’ of mitomycin [14]. A summary of standard (Arm A) and experimental (Arm B) treatment regimens can be seen in Fig. 2.

### Standard treatment group: BCG alone (Arm A)

Standard intravesical therapy with BCG alone consists of induction (BCG once a week for six weeks) followed by four-weekly maintenance starting at week 13 (weeks 13, 17, 21, 25, 29, 33, 37, 41, 45, and 49). BCG (either 2–8 x 10^8^ CFU for OncoTICE™, or 81 mg for ImmunoCYST™ and TheraCys™) is reconstituted with sterile water then diluted in 50 mL of 0.9 % saline. The same brand of BCG is used for all treatment administered to an individual participant throughout the study.

### Experimental treatment group: BCG and mitomycin (Arm B)

Experimental intravesical therapy with BCG and mitomycin consists of induction (BCG once a week for weeks 1, 2, 4, 5, 7, and 8; mitomycin once a week for weeks 3, 6, and 9) followed by four-weekly maintenance starting at week 13 (mitomycin weeks 13, 17, 25, 29, 37, and 41; BCG weeks 21, 33, and 45). Mitomycin (40 mg) is reconstituted and diluted in 40 mL of sterile water; BCG is dosed, reconstituted, and brand-nominated as per Arm A.

### Delivery, modification, and discontinuation of treatment

Intravesical treatment is administered in a standard way with the agent being maintained within the bladder for approximately one to two hours. Participants are screened for urinary tract infection (UTI) prior to the commencement of both induction and maintenance treatment; additional testing is performed during treatment based on clinical suspicion. No treatment is delivered within seven days of a TURBT or bladder biopsy, or in the presence of UTI or significant treatment-related toxicity. Treatment delays of up to four weeks during induction, and twelve weeks during maintenance, are permitted before the remaining induction or maintenance doses are omitted. Dose reduction is not permitted for mitomycin, however the dose of BCG can be permanently reduced to a third of the normal dose at the discretion of the treating clinician. Treatment is discontinued in the event of unacceptable toxicity, excessive treatment delay, disease progression, recurrent high-grade T1 tumours, or persistent high-grade or T1 tumours despite induction and four maintenance instillations.

### Outcomes and measures

The primary outcome is disease-free survival (DFS). Secondary outcomes are disease activity, time to recurrence, time to progression, overall survival, adverse events (assessed using NCI CTCAE v4.0), health-related quality of life (assessed using QLQBLS-24 [19], QLQ-C30 [20], I-PSS [16]), feasibility (proportion of participants receiving at least 75 % of planned instillations), and resource use (inpatient admissions, outpatient appointments, general practitioner and emergency department visits). The schedule of assessments that occur in the absence of progression is summarised in Table 1.

**Table 1:** Schedule of assessments

	Baseline	Induction	Maintenance	Year 2 to Year 5
**Blood tests** ^**a**^	X		Week 13	
**Urine culture**	X		Week 13	
**Cystoscopy** ^**b**^	TURBT	After last instillation	After 4^th^, 7^th^ and last instillation	3 monthly (Year 2); 6 monthly (Years 3 and 4); at 5 years
**Health-related quality of life**	X	Weeks 3,6,9,13	Weeks 25,37,49	3 monthly (Year 2); 6 monthly (Years 3 and 4); at 5 years
**Assessments** ^**c**^	X	Prior to each treatment	Prior to each treatment	3 monthly (Year 2); 6 monthly (Years 3 and 4); at 5 years

^a^Full blood count, urea and electrolytes, liver function tests

^b^Under local anaesthesia (LA) except at end of induction and maintenance when must be under general anaesthesia (GA) with biopsies

^c^Assessments include those for treatment toxicity, symptoms of UTI, adverse events, resource use and patient status

### Biological and translational sub-studies

Participants provide written informed consent for donation of formalin-fixed paraffin-embedded tumour tissue that has already been collected, for use in future biological or translational sub-studies. These studies aim to identify biomarkers associated with prognosis or response to treatment.

### Ethical considerations

The protocol gained central ethics approval in June 2013. The study is conducted in accordance with the Declaration of Helsinki, Note for Guidance on Good Clinical Practice (CPMP/ICH/135/95 - annotated with TGA comments), NHMRC National Statement on Ethical Conduct in Human Research, NHMRC Australian Code for the Responsible Conduct of Research, and all applicable laws and regulations.

### Statistical considerations

The planned sample size of 500 participants (250 per arm), recruited over four years and followed for another two years, is designed to give 85 % power to detect a 37 % reduction in the hazard for DFS (hazard ratio 0.63, corresponding to a two-year DFS probability of 70 % [BCG alone] vs. 80 % [BCG and mitomycin) at the 5 % level of statistical significance allowing for a non-compliance rate of 10 %. Under these assumptions, 213 DFS events (progression or death) are expected to be observed. The anticipated two-year DFS probability for the control group (BCG alone, 70 %) is based on results with single-agent BCG with maintenance in previous randomised trials and meta-analyses [5, 11, 14].

### Analysis plan

The primary analysis will be a comparison of treatment groups on DFS using a log-rank test; Kaplan-Meier curves will also be used to estimate DFS at two years. Cox proportional hazards regression and modelling will be used to estimate a hazard ratio for the treatment effect on DFS before and after adjusting for covariates. Similar analysis will be conducted of other time to event endpoints including time to recurrence, time to progression, and overall survival. Comparisons on categorical and continuous outcomes will be undertaken using suitable non-parametric and regression models, and will adjust for covariates. Formal cost-effectiveness analyses will be conducted if the main endpoints warrant consideration of a change in practice.

Additionally, three interim analyses will be conducted; once the first 130 participants complete treatment, and after a third and two thirds of the required number of DFS events have occurred. The interim analyses will assess feasibility, adverse events, study performance, and preliminary DFS data to determine whether the trial should stop, be modified, or continue unchanged; these analyses will be reviewed by an Independent Data and Safety Monitoring Board.

## Discussion

This trial aims to determine whether the addition of intravesical chemotherapy to adjuvant BCG treatment reduces the risk of recurrence and progression of high-risk NMIBC, over treatment with BCG alone. Our meta-analysis suggests that combination treatment may be more effective [13], however the only individual study to prove a benefit did not include people with Ta disease and delivered mitomycin via unconventional ‘electromotive delivery’ [14]. If this trial confirms the benefits of combination intravesical therapy with BCG and mitomycin, over BCG alone, in all high-risk NMIBC subgroups when delivered in a standard way then the implications of this on the treatment of NMIBC worldwide would be significant. This trial will assess the comparative efficacy of these two treatments by collecting outcome data including DFS, disease activity, time to recurrence, time to progression, and overall survival. The widespread adoption of combination treatment with BCG and mitomycin will also be affected by its tolerability and side-effects as compared to treatment with BCG alone. The comparative tolerability and side-effects of the two treatments will be assessed in this trial by collecting data on adverse events, health-related quality of life, feasibility, and resource use.

Additionally, the consent process for this trial invites participants to consent to use of formalin-fixed, paraffin-embedded tumour tissue that is collected, for use in biological or translational sub-studies. These studies may potentially provide a better understanding of how BCG and mitomycin work in NMIBC, or identify biomarkers that are able to better predict success of intravesical treatment or overall prognosis.

## Abbreviations

ANZUP: Australian and New Zealand Urogenital and Prostate Cancer Trials Group
BCG: Bacillus Calmette-Guérin
CFU: Colony forming units
CIS: Carcinoma-in-situ
DFS: Disease-free survival
ECOG: Eastern Co-operative Oncology Group
EORTC: European Organisation for Research and Treatment of Cancer
GA: General anaesthesia
I-PSS: International Prostate Symptom Score
LA: Local anaesthesia
MRC: Medical Research Council
NCI CTCAE: National Cancer Institute Common Terminology Criteria for Adverse Events
NHMRC: National Health and Medical Research Council
NMIBC: Non-muscle-invasive bladder cancer
QLQ-BLS24: Quality of life questionnaire for bladder cancer that is superficial (24 items)
QLQ-C30: Quality of life questionnaire core 30
TURBT: Trans-urethral resection of the bladder tumour
UTI: Urinary tract infection
